# Personalized Medicine in Pulmonary Arterial Hypertension: Utilizing Artificial Intelligence for Death Prevention

**DOI:** 10.3390/jcm14238325

**Published:** 2025-11-23

**Authors:** Łukasz Ledziński, Grzegorz Grześk, Michał Ziołkowski, Marcin Waligóra, Marcin Kurzyna, Tatiana Mularek-Kubzdela, Anna Smukowska-Gorynia, Ilona Skoczylas, Łukasz Chrzanowski, Piotr Błaszczak, Miłosz Jaguszewski, Beata Kuśmierczyk-Droszcz, Katarzyna Ptaszyńska, Katarzyna Mizia-Stec, Ewa Malinowska, Małgorzata Peregud-Pogorzelska, Ewa Lewicka, Michał Tomaszewski, Wojciech Jacheć, Michał Florczyk, Ewa Mroczek, Zbigniew Gąsior, Agnieszka Pawlak, Katarzyna Betkier-Lipińska, Piotr Pruszczyk, Olga Dzikowska-Diduch, Katarzyna Widejko, Judyta Winowska-Józwa, Grzegorz Kopeć

**Affiliations:** 1 Department of Cardiology and Clinical Pharmacology, Collegium Medicum in Bydgoszcz, Nicolaus Copernicus University in Toruń, 85-168 Bydgoszcz, Poland; 503345@doktorant.umk.pl (Ł.L.); boldynski@wp.pl (M.Z.); 2 Department of Cardiac and Vascular Diseases, St. John Paul II Hospital in Krakow, 31-202 Krakow, Poland; 3 Pulmonary Circulation Centre, Department of Cardiac and Vascular Diseases, Faculty of Medicine, Jagiellonian University Medical College, 31-202 Krakow, Poland; 4 Center for Innovative Medical Education, Department of Medical Education, Faculty of Medicine, Jagiellonian University Medical College, 30-688 Krakow, Poland; 5 Department of Pulmonary Circulation, Thromboembolic Diseases and Cardiology, Centre of Postgraduate Medical Education, Fryderyk Chopin Hospital in European Health Centre Otwock, 05-400 Otwock, Poland; marcin.kurzyna@ecz-otwock.pl (M.K.); michal.florczyk@ecz-otwock.pl (M.F.); 6 Department of Cardiology, Poznan University of Medical Sciences, 61-701 Poznan, Poland; 7 3rd Department of Cardiology, Faculty of Medical Sciences in Zabrze, Medical University of Silesia, 41-800 Katowice, Poland; 8 Cardiology Department, Medical University of Lodz, 91-347 Lodz, Poland; chrzanowski@ptkardio.pl; 9 Department of Cardiology, Cardinal Wyszynski Hospital, 20-718 Lublin, Poland; 10 1st Department of Cardiology, Medical University of Gdańsk, 80-210 Gdańsk, Poland; 11 Department of Congenital Heart Disease, Institute of Cardiology, 04-628 Warsaw, Poland; 12 Department of Cardiology, Medical University of Bialystok, 15-276 Bialystok, Poland; 13 Centre of the European Reference Network for Rare, Low Prevalence or Complex Diseases of the Heart (ERN GUARD Heart), First Department of Cardiology, School of Medicine in Katowice, Medical University of Silesia in Katowice, 40-635 Katowice, Poland; 14 Pulmonary Department, University of Warmia and Mazury, 10-357 Olsztyn, Poland; 15 Department of Cardiology, Pomeranian Medical University in Szczecin, 70-111 Szczecin, Poland; malgorzata.peregud.pogorzelska@pum.edu.pl; 16 Department of Cardiology and Electrotherapy, Medical University of Gdansk, 80-211 Gdansk, Poland; elew@gumed.edu.pl; 17 Department of Cardiology, Medical University of Lublin, 20-090 Lublin, Poland; 18 2nd Department of Cardiology, Faculty of Medical Sciences in Zabrze, Medical University of Silesia in Katowice, 41-800 Zabrze, Poland; 19 Clinic of Heart Diseases, Institute of Heart Diseases, University Clinical Hospital in Wrocław, 50-556 Wrocław, Poland; 20 Department of Cardiology, School of Health Sciences in Katowice, Medical University of Silesia in Katowice, 40-635 Katowice, Poland; 21 Department of Cardiology, National Medical Institute of the Ministry of the Interior and Administration, 02-507 Warszawa, Poland; a.pawlak1@wp.pl; 22 Clinical and Research Department of Applied Physiology, Mossakowski Medical Research Institute, Polish Academy of Sciences, 02-106 Warszawa, Poland; 23 Department of Cardiology and Internal Medicine, Military Institute of Medicine—National Research Institute, 04-141 Warsaw, Poland; kbetkier-lipinska@wim.mil.pl; 24 Department of Internal Medicine and Cardiology with the Center for Diagnosis and Treatment of Venous Thromboembolism, Medical University of Warsaw, 02-005 Warszawa, Poland; piotr.pruszczyk@wum.edu.pl (P.P.); olga.dzikowska-diduch@wum.edu.pl (O.D.-D.); 25 Department of Cardiology, Copper Health Center, 59-300 Lubin, Poland; 26 Department of Cardiology, Provincial Specialist Hospital in Szczecin, 70-111 Szczecin, Poland; j.winowska@gmail.com

**Keywords:** pulmonary arterial hypertension (PAH), machine learning, XGBoost, SHAP, mortality prediction, personalized medicine, risk stratification

## Abstract

**Background/Objectives**: Pulmonary arterial hypertension (PAH) is a complex cardiovascular disease with a high burden of morbidity and mortality. Although several risk prediction models have been proposed, the exact significance of distinct clinical parameters in predicting survival in PAH remains unclear. It is important to emphasize that this study does not aim to validate or contradict existing clinical risk assessment calculators provided by the ESC or other scientific societies. Instead, the goal of this research is to identify and rank clinical parameters according to their importance in predicting mortality in PAH patients using machine learning techniques. **Methods**: Using the Database of Pulmonary Hypertension in the Polish population (BNP-PL) registry, 1755 adult patients with PAH were selected. Feature engineering was conducted using domain knowledge, guided by European Society of Cardiology (ESC) recommendations. Features were reduced using LASSO regression and sequential feature elimination algorithms. A classification model was built using the XGBoost algorithm, utilizing 17 features. The model was tested on a preselected subset of the BNP-PL data. The Shapley Additive Explanations (SHAP) method was used to explain the model’s predictions and to rank feature importance. **Results**: The model achieved satisfactory results across evaluated metrics, including an area under the curve of 0.767, accuracy of 0.738, specificity of 0.733, and sensitivity of 0.800. SHAP values effectively ranked the features, corroborating the significance of parameters present in the ESC risk stratification tables. Furthermore, local interpretation of results using SHAP enabled individualized assessment of feature importance, enhancing clinical applicability. **Conclusions**: The proposed artificial intelligence-based model demonstrates satisfactory predictive capability, highlighting the potential of machine learning techniques to support more personalized approaches to the management of PAH patients. This approach offers complementary insights into traditional risk assessment methods, providing clinicians with a novel tool for individualized risk evaluation and decision-making.

## 1. Introduction

Pulmonary arterial hypertension (PAH) is a rare, multifactorial cardiovascular disease characterized by increased resistance of the pulmonary arteries due to the proliferation of vascular cells. Over time, the elevated pressure in the pulmonary circulation leads to cardiac overload, remodeling, and ultimately right heart failure, with significantly increased risk of mortality [1].

Early diagnosis and intervention are critical for enhancing patients’ prognosis and survival. Predicting unplanned deaths in particular patients suffering from PAH is a significant challenge.

The application of machine learning (ML) models allows for the early identification of patients at increased risk of death, enabling better management of their therapy and more effective medical interventions. Importantly, for the healthcare system, this also aids in optimizing the costs of patient care. ML models can analyze complex relationships between various risk factors, such as functional test results, medical history, pharmacotherapy, and patient lifestyle, providing satisfactory predictions.

Recent years have witnessed a rapid expansion in the use of artificial intelligence (AI) and ML across various domains of cardiology, including risk stratification, arrhythmia detection, and outcome prediction. Large-scale studies have demonstrated that ML-based approaches can outperform traditional statistical methods in predicting adverse events and mortality, especially in complex, multifactorial diseases such as PAH. The integration of AI-driven models into clinical workflows enables the synthesis of high-dimensional data from electronic health records, imaging, and laboratory results, facilitating personalized medicine and more precise prognostication. The utilization of artificial intelligence in cardiology spans multiple aspects. This includes image processing, natural language processing, and wearable devices used by patients daily [2,3,4,5,6]. This trend is driven by both the desire of patients and physicians to leverage the latest technologies and the academic community’s pursuit of current trends.

In this context, our study seeks to bridge this gap by leveraging state-of-the-art ML techniques to develop a predictive model for mortality in adult PAH patients. By combining clinical expertise with advanced data analytics, we aim to contribute to the growing body of evidence supporting the use of AI in cardiovascular medicine and to provide clinicians with practical tools for improving patient outcomes.

The aim of this research is to identify risk factors associated with mortality among PAH patients and to develop a machine learning model capable of accurately predicting death. As a tool, it will support clinicians in making informed decisions, optimizing treatment strategies, and ultimately improving patient care and outcomes. According to our current knowledge, the application of ML specifically to mortality prediction in adult PAH patients remains limited in the literature.

## 2. Materials and Methods

The study utilized the Database of Pulmonary Hypertension in the Polish population (BNP-PL) registry, which contains examination results of PAH patients collected from medical centers across Poland. BNP-PL is the first multicenter prospective registry of adult and pediatric [7] patients with PAH and patients with Chronic Thromboembolic Pulmonary Hypertension (CTEPH) [8] created in Central and Eastern European countries [9]. The methodology of BNP-PL has been previously described [10].

The data obtained from the BNP-PL registry were initially cleaned, and subsequently, 165 features were extracted for 1755 adult patients with PAH. From the baseline visit, information on height, weight, blood pressure, and demographic details was selected. Additionally, detailed data from laboratory tests, right heart catheterization, functional tests, and echocardiography were extracted. An important element was the collected medical history, which included information on current comorbidities, ongoing treatments, and past medical procedures. From the follow-up part of the database, information on death due to sudden cardiac death, respiratory failure, acute pulmonary embolism, acute coronary syndrome, and progression of heart failure from baseline assessment to the first follow-up was obtained.

The initially selected data was then preprocessed by removing extreme outliers and duplicates.

As part of feature engineering, the stroke volume index and the tricuspid annular plane systolic excursion (TAPSE) to systolic pulmonary arterial pressure (sPAP) ratio were calculated. Additionally, the number of diuretics taken was encoded.

All categorical variables were encoded using one-hot encoding, except for the variable “Use of diuretics,” whose value reflects the number of different diuretic medications used concurrently. The diuretics used in the BNP-PL population included furosemide, torasemide, thiazide diuretics, and aldosterone antagonists. The age of patients was binned into 5-year intervals and ordinally encoded. The target variable, which was mortality, was binarily encoded. Next, feature selection was performed using the Least Absolute Shrinkage and Selection Operator (LASSO) algorithm, followed by sequential feature elimination (SFE). While each method independently identifies relevant predictors, their consecutive use may appear methodologically redundant. The rationale for this approach was to leverage the strengths of LASSO in penalizing less informative features and SFE in iteratively refining the feature set based on model performance. Specifically, LASSO was first used to reduce the initial feature pool by discarding 140 features with coefficients shrunk to zero. Subsequently, SFE further eliminated 8 features by evaluating their impact on classification accuracy. This two-step process aimed to enhance model generalizability and interpretability by ensuring that only the most robust predictors were retained. The selected dataset was then split into train and test sets using StratifiedKFold in an 80:20 ratio to ensure that both sets maintained the same class distribution, which is particularly important for imbalanced datasets. An 80:20 split ratio was chosen to maximize the amount of data available for training the model [11].

The resulting datasets had target variable ratios of 1303:101 and 326:25 for the train and test sets, respectively. The test set was reserved for the final evaluation of the model’s quality and was not used during the training, validation, and optimization processes of the trained model.

To prepare the machine learning model, tree-based classifiers were planned for use due to their ease of interpretation, which is crucial for the potential use of the results as a clinical support tool for specialist doctors. The eXtreme Gradient Boosting (XGBoost 2.1.3) algorithm is a powerful and efficient implementation of the gradient boosting framework, widely used for supervised learning tasks such as classification and regression [12]. It has gained popularity due to its high performance, scalability, and flexibility. It has been successfully applied in numerous data science competitions and real-world applications, demonstrating its effectiveness and versatility. XGBoost was chosen for its ability to handle missing data, computational speed, ease of optimization, and the possibility of explaining predictions using the Shapley Additive Explanations (SHAP) values [13,14]. The entire training process was conducted using RepeatedStratifiedKFold cross-validation (n_splits = 10, n_repeats = 5) to ensure robustness and accuracy of the results. Hyperparameter tuning was conducted using the HyperOpt framework with cross-validation, the same as in the training process [15]. During hyperparameter optimization using the hyperopt library, the following hyperparameters were tuned: colsample_bytree, gamma, learning_rate, max_depth, n_estimators, min_child_weight, reg_alpha, reg_lambda, scale_pos_weight, and subsample. Due to the high class imbalance in the training dataset, various oversampling methods such as SMOTE and ADASYN, as well as the combined over- and under-sampling method SMOTETomek, were tested [16]. However, these approaches did not yield satisfactory results, so only hyperparameter optimization was ultimately used.

The fine-tuned and trained model was used to make predictions on the test set, the results of which are presented later in this work. SHAP values were used to achieve explainable artificial intelligence (XAI). SHAP values are based on the concept of Shapley values from cooperative game theory, measuring the contribution of each feature to the model’s prediction by considering all possible combinations of features. Using this interpretative technique allows for the identification of the most important features, thereby indicating risk factors both globally and for each individual patient case.

**Figure 1:**
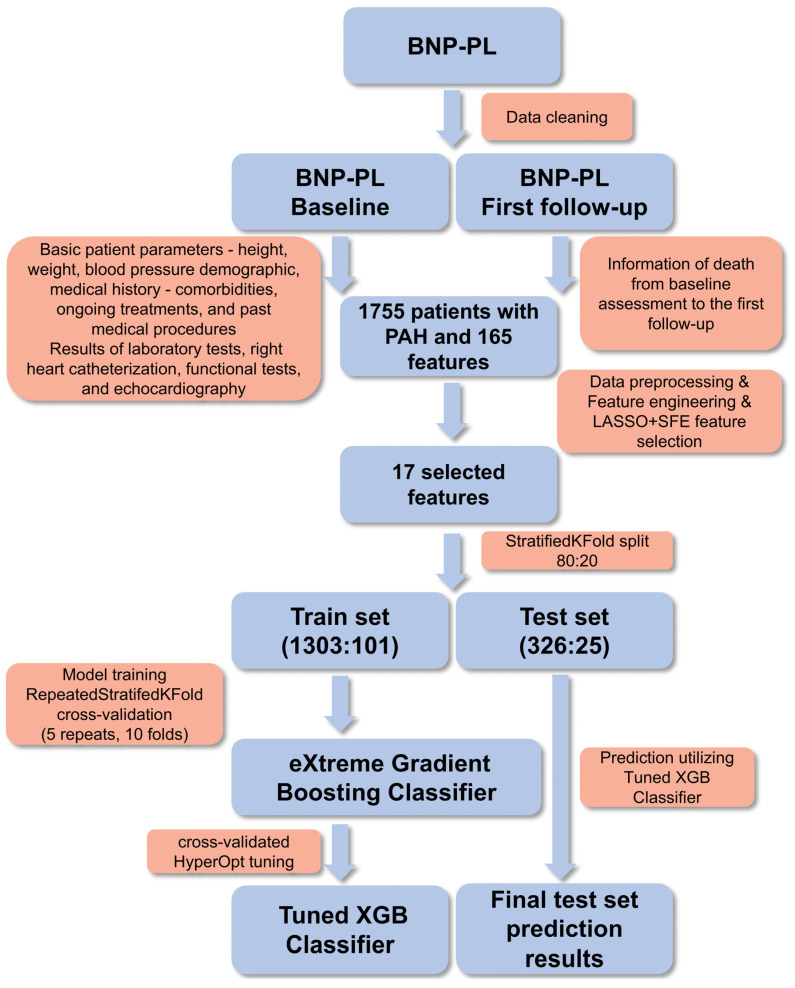
Study workflow. BNP-PL—Database of Pulmonary Hypertension in the Polish population; LASSO—Least Absolute Shrinkage and Selection Operator regression; PAH—Pulmonary Arterial Hypertension; SFE—Sequential Feature Elimination; XGB—eXtreme Gradient Boosting.

The entire workflow is presented in Figure 1.

To evaluate the quality of the model, typical metrics commonly used for binary classification assessment and the confusion matrix were employed.

The confusion matrix is a summary table of the classification results of the model, containing:

- TP (True Positive): Correctly predicted positive cases.
- TN (True Negative): Correctly predicted negative cases.
- FP (False Positive): Incorrectly predicted positive cases.
- FN (False Negative): Incorrectly predicted negative cases.

These metrics provide a comprehensive view of the model’s performance.

Accuracy represents the percentage of correctly classified samples out of all samples. It indicates the overall quality of the model and is very intuitive to interpret. However, for imbalanced datasets, accuracy can be misleading, as it may not reflect the model’s performance on the minority class.

(1) $$Accuracy=\frac{TP+TN}{TP+TN+FP+FN},$$

Specificity measures the ability to correctly classify the negative class. It is important in scenarios where the cost of Type I errors (false positives) is high and needs to be minimized. High specificity ensures that most actual negative cases are correctly identified by the model.

(2) $$Specificity=\frac{TN}{TN+FP},$$

Sensitivity, also known as recall, measures the ability to correctly classify the positive class. It is crucial in scenarios where the cost of Type II errors (false negatives) is high and needs to be minimized. High sensitivity ensures that most actual positive cases are identified by the model.

(3) $$Sensitivity=\frac{TP}{TP+FN},$$

The Matthews Correlation Coefficient (MCC) is a balanced measure of binary classification quality that evaluates all four values of the confusion matrix. It is particularly reliable for imbalanced datasets. The MCC value ranges from −1 to +1, where +1 indicates a perfect prediction, 0 indicates no better than random prediction, and −1 indicates total disagreement between prediction and observation.

(4) $$MCC=\frac{\left(TP\cdot TN\right)-(FP\cdot FN)}{\sqrt{(TP+FP)\cdot (TP+FN)\cdot (TN+FP)\cdot (TN+FN),}}$$

The Receiver Operating Characteristic—Area Under the Curve (ROC-AUC) is the area under the curve that represents the relationship between sensitivity and 1-specificity at various decision thresholds. It evaluates the model’s ability to distinguish between classes; the higher the value, the better the model’s ability to predict the positive class.

For the purpose of the present analysis, we defined extended hospitalization for patients with PAH, which refers to hospital stays longer than the average duration for patients with this condition. This duration can vary between hospitals contributing data to the database due to differences in patient populations with the same diagnosis.

All calculations were performed in a Python 3.10.12 environment using publicly available libraries. The computations were conducted on a computer with a CPU featuring 24 threads at 4.5 GHz and 64 GB of RAM. Packages versions: pandas (2.1.4), numpy (1.26.4), hyperopt (0.2.7), shap (0.46.0), scikit-learn (1.4.2), xgboost (2.1.3).

Categorical variables were expressed as counts and compared using the chi-square test. Continuous variables were presented as mean ± standard deviation (SD) or median and first and third quartiles (Q1, Q3). Continuous variables were analyzed using the U-Mann–Whitney test. A two-tailed test result with a *p*-value < 0.05 was considered statistically significant.

## 3. Results

The general characterization of patients with PAH in the BNP-PL registry was presented previously by Kopeć et al. [9]. Between 1 March 2018 and 31 Aug 2023, a total of 1755 PAH patients were enrolled including 902 newly diagnosed and 853 previously (before 1 March 2018). At the same time, 126 patients died. The median/mean time from baseline visit to death was 10 and 12 months respectively. The study cohort included a diverse range of PAH etiologies. The most common type was idiopathic non-reactive PAH (750 patients, 8% non-survivors), followed by PAH associated with congenital heart disease (483 patients, 5% non-survivors) and PAH associated with connective tissue diseases (305 patients, 10% non-survivors). Less frequent types included idiopathic reactive PAH (121 patients, 2% non-survivors), PAH associated with portal hypertension (45 patients, 7% non-survivors), heritable PAH (19 patients, 5% non-survivors), PAH associated with HIV infection (10 patients, 0% non-survivors), and PAH induced by drugs or toxins (14 patients, 14% non-survivors). Pulmonary veno-occlusive disease was rare (6 patients) but had the highest mortality rate (33%). Only two cases were classified as unknown etiology. The baseline characteristics of patients with PAH were compared in relation to mortality due to sudden cardiac death, respiratory failure, acute pulmonary embolism, acute coronary syndrome, and progression of heart failure in the PAH patient population.

Table 1 below summarizes the statistics of continuous and categorical variables selected by the LASSO regression and SFE used to build the XGB classification model.

The classification model based on the XGBoost algorithm demonstrates moderate performance on the test data. While the model maintains good sensitivity and a low rate of false negatives, it exhibits a relatively high proportion of false positive predictions, which translates into low precision. Table 2 below summarizes the key performance metrics for the model. These metrics indicate the model’s effectiveness in predicting death by the next follow-up in the population of Polish patients with PAH.

Particular attention should be given to the sensitivity of the predictive model, which, considering the significant class imbalance, was made possible through the optimization parameters available for the XGBoost algorithm. Table 3 contains the confusion matrix for the test set.

**Figure 2:**
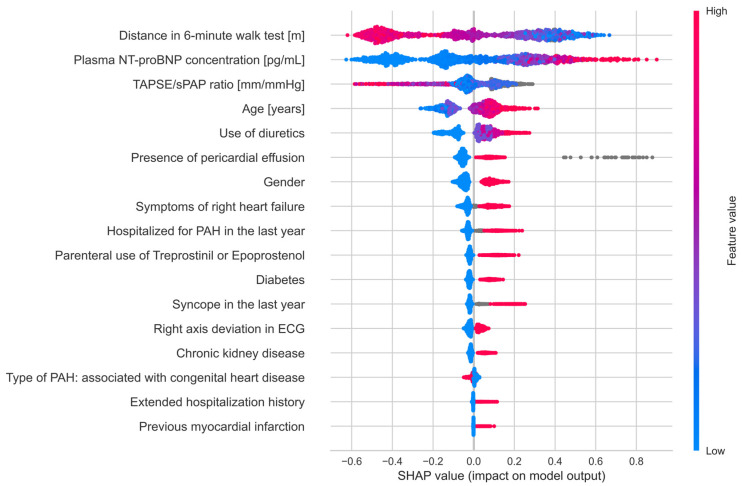
Distribution of SHAP values for all 17 features. The color gradient indicates the actual feature values, with blue representing lower values and red representing higher values, and gray representing missing values. The horizontal spread of points for each feature indicates the variability in the SHAP values, reflecting the feature’s influence on the model’s predictions. A wider vertical spread of points for a feature suggests a larger number of observations or greater diversity in the feature’s values.

**Figure 3:**
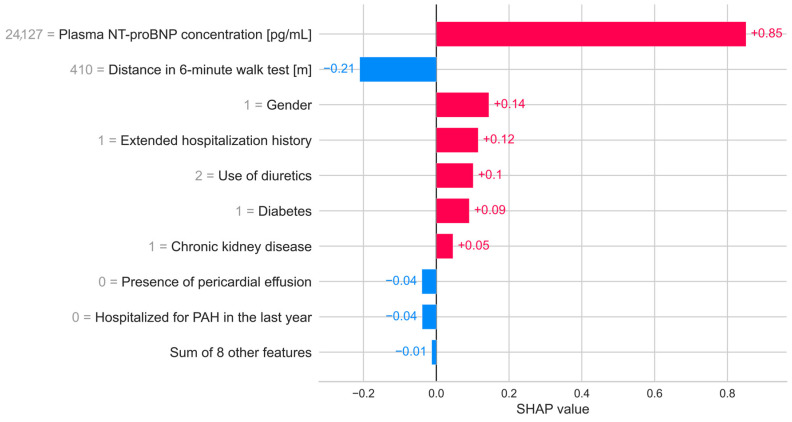
The bar plot of the top nine features illustrates the SHAP values for a specific patient, highlighting the contribution of each feature to the model’s prediction. Each bar represents a feature, with the length of the bar indicating the magnitude of the feature’s impact on the prediction. Positive SHAP values (bars extending to the right) indicate features that increase the risk of death, while negative SHAP values (bars extending to the left) indicate features that decrease the risk of death. The label on the Y-axis corresponds to the actual values of the features for this patient. This detailed breakdown allows for a clear understanding of the key factors driving the model’s decision for this particular patient. In this case, the model predicted the patient as a non-survivor, and indeed, the patient belonged to the non-survivor group.

The satisfactory results presented by performance metrics indicate that the use of the XGBoost algorithm to predict death by the next follow-up in the population of Polish patients with PAH is fully justified. The features indicated by SHAP values highlight significant clinical parameters that can be used for risk assessment and modification of patient treatment. The value of feature engineering in improving the model’s quality is noteworthy, as evidenced by the high SHAP values for features prepared based on domain knowledge. The SHAP “beeswarm” plot represents the distribution of SHAP values for each feature, showing the variability in feature impact across different instances. This visualization helps to understand how different feature values influence the model’s predictions (Figure 2). The features identified by the model are reflected in the ESC guidelines for stratifying patients into risk groups, including blood NT-proBNP concentration, 6-min walk test result, TAPSE to sPAP ratio [17,18], and the presence of pericardial effusion. All these parameters are considered to have the greatest impact on predictive ability, as expressed by SHAP values. Locally indicating the most important features allows for easy interpretation and potential medical intervention (Figure 3). Among the features that proved to be most significant for the predictive model were symptoms of right ventricular failure, TAPSE to sPAP ratio, 6MWT, presence of pericardial fluid, NT-proBNP serum concentration, right axis deviation in ECG, age, gender, comorbidities—diabetes and chronic kidney disease—and medications used in PAH therapy or to treat comorbidities. These medications include prostacyclin analog treprostinil and epoprostenol, diuretics such as torasemide and aldosterone antagonists or thiazide diuretics.

## 4. Discussion

The obtained results provide hope for the development of a tool for cardiologists that allows for the optimization of treatment for patients with PAH. Such an approach may not only enable personalized treatment but also potentially improve patients’ prognosis [19,20].

The most closely related study in terms of its assumptions, predicting 30-day readmission in pediatric pulmonary hypertension, utilized the CatBoost algorithm, which belongs to the group of gradient boosting algorithms [21]. The authors of the study achieved very good results with an AUC of 0.81, high accuracy of 0.74, sensitivity of 0.78, and specificity of 0.74. However, due to the different patient population and the limitation of prediction to 30-day readmission, direct comparison of the obtained results is not possible. Another example of a related study is the research that utilized machine learning to predict one-year mortality in newly diagnosed patients with PAH. The authors, using data from cardiac magnetic resonance imaging, achieved very satisfactory results, improving the REVEAL score predictions from a c-index of 0.71 to 0.76. However, fundamental differences in methodological approach and the type of data used do not allow for a direct comparison of the results obtained in this study with those of the present work [22]. The lack of studies with assumptions more closely aligned to those presented in this research does not allow for comparison of the results obtained by other researchers [23,24,25,26]. Moreover, the recent literature highlights the successful application of artificial intelligence in various fields of cardiology, including arrhythmia management and cardiac electrophysiology. For example, Cersosimo et al. demonstrated that AI-based models, such as ChatGPT-4o, can support ECG interpretation, arrhythmia detection, procedural guidance during ablation, and risk stratification for sudden cardiac death. These advances illustrate the transformative potential of AI in improving diagnostic accuracy, optimizing clinical workflows, and personalizing therapy. However, the authors also emphasize the importance of balancing AI adoption with clinical expertise and ethical considerations, underscoring the need for collaboration between clinicians and AI developers to ensure reliable and transparent solutions in cardiovascular care [27].

A comparative analysis was performed between gradient boosting frameworks such as LightGBM, CatBoost, and AdaBoost, alongside XGBoost. Although LightGBM and CatBoost are known for their computational efficiency and advanced handling of categorical variables, and AdaBoost for its simplicity, our experiments showed that these algorithms consistently yielded inferior predictive performance compared to XGBoost on our dataset. Additionally, given the relatively small size of our dataset, differences in computation time between these frameworks were negligible. To maintain clarity and focus, only XGBoost results were included in the final manuscript. The choice of XGBoost was further justified by its robust regularization capabilities, scalability, and proven effectiveness in handling clinical tabular data, which are critical for PAH mortality prediction tasks. This approach aligns with current literature, where XGBoost remains a leading choice for biomedical machine learning applications.

It is important to emphasize that the SHAP values indicating the significance of medications used by patients do not point to the medication itself, which is used according to the treatment guidelines for PAH patients. Instead, they reflect the clinical condition of the patient that necessitates appropriate pharmacological treatment. The presented model serves as a classifier indicating the likelihood of a patient’s mortality before the next follow-up, rather than specifying the exact therapeutic steps to avoid such outcomes. The identification of medications as significant predictors does not imply a need to alter their usage but highlights the necessity for closer monitoring of patients’ health status, as clinical deterioration may occur independently of the medication itself (e.g., diuretics). Moreover, parenteral prostacyclins such as treprostinil or epoprostenol are typically reserved for patients with more advanced disease, serving as an indicator of baseline severity rather than a direct contributor to increased mortality risk [28]. Conversely, medications that modulate heart rate, such as beta-blockers or ivabradine, which are used to counteract hyperadrenergic drive in heart failure, may adversely affect survival in PAH and are generally contraindicated unless justified by specific comorbidities [29]. The presence of diabetes among the highly significant features is quite understandable. Patients with PAH and diabetes present with more advanced pulmonary vascular disease and worse survival rates compared to their counterparts without diabetes [30].

The demonstrated significant features may require confirmation in clinical studies and serve as a kind of guideline for what should be investigated in multicenter clinical trials, especially in the context of increased risk of readmission or death. Many of the predictive factors indicated by the model are complex and multifactorial, making them difficult to interpret unequivocally. For example, prolonged hospitalizations, where the reason for the extension is unknown, cannot be conclusively attributed solely to the underlying disease, PAH. It might have been influenced by factors independent of the patient, such as the occurrence of an infection or an injury that required the patient to remain under observation in the hospital. The prepared model has its limitations due to the available data. It would be necessary to train on a larger dataset, preferably containing information about patients from multiple populations. A dataset containing data only from one country may be biased by the healthcare funding system, which affects the medications used, significantly impacting the model’s predictive capabilities. The limitations in the number of patients in the database and bias towards a specific population also affect the model’s stability and the risk of poor prediction on entirely external datasets. Another problem is the unequal time from baseline assessment to follow-up among patients. Introducing a fixed time interval for follow-up registration would significantly improve the applicability of machine learning.

Most current research focuses on diagnostic algorithms in PAH. Nonetheless, technologies such as gradient boosting and feature importance explanation using SHAP are also prevalent in these studies [31,32,33,34]. The utilization of explainable machine learning models appears to be essential in the field of medicine [35].

Additionally, there is a noticeable need for the creation of multidisciplinary research teams where clinicians can provide their domain expertise, and data scientists can use this knowledge to build better models that yield satisfactory results and are more robust. The development of a personalized approach to patient treatment in the future will require us to utilize modern technologies based on artificial intelligence, whose advancement has significantly accelerated in recent years [36].

Despite satisfactory results, the presented machine learning model still leaves a considerable margin of error, especially when viewed through the lens of human health and life. One of the next steps in the development of similar studies should be to investigate the impact of PAH type on the predictive capabilities of the model. The presented model has considerable issues with precision, resulting in a high rate of false positive predictions, as shown in Table 3. However, we believe that drawing attention to a patient whose actual risk of death is lower is a lesser problem than missing a patient with a genuinely high risk of death. From an ethical and moral standpoint, this approach seems more appropriate. Certainly, the next stage of our future work will be to search for a model that maintains both a low rate of false positive and false negative predictions. However, to achieve this, it is necessary to first address the remaining limitations. AI solutions facilitate and accelerate the work of physicians, but decision-making should always be critically evaluated through the lens of clinical experience [37].

## 5. Conclusions

The application of machine learning to predict mortality in patients with PAH yields satisfactory results. However, further research based on large international registries of this rare disease is required.

In the future, a tool similar to this could facilitate work in every hospital and provide invaluable support in the daily challenges of caring for patients with PAH. The integration of AI-driven predictive analytics in PAH management holds immense promise for revolutionizing clinical decision-making and improving patient outcomes. By leveraging advanced machine learning algorithms and comprehensive patient data, healthcare providers can identify high-risk individuals, customize treatment approaches, and implement proactive interventions to prevent mortality.

## Figures and Tables

**Table 1:** Baseline characteristics of PAH patients in the BNP-PL registry in relation to survival status. The presented variables were chosen in the feature selection step.

Variable	Survivors (n = 1629)	Non-Survivors (n = 126)	*p*-Value
Age [years], median (Q1,Q3)	59 (40, 70)	68 (57.75, 74)	0.0000
Gender, male, n (%)	509 (31.25%)	51 (40.48%)	0.0411
Type of PAH: associated with congenital heart disease, n (%)	485 (28.12%)	25 (19.84%)	0.0574
Chronic kidney disease, n (%)	274 (16.82%)	45 (35.71%)	0.0000
Diabetes, n (%)	322 (19.77%)	45 (35.71%)	0.0000
Previous myocardial infarction, n (%)	78 (4.79%)	21 (16.67%)	0.0000
Hospitalized for PAH in the last year, n (%)	240 (15.81%)	37 (31.36%)	0.0000
Extended hospitalization history, n (%)	140 (8.59%)	21 (16.67%)	0.0042
Syncope in the last year, n (%)	97 (6.39%)	18 (15.25%)	0.0006
Symptoms of right heart failure, n (%)	493 (32.48%)	75 (63.56%)	0.0000
Right axis deviation in ECG, n (%)	626 (38.43%)	69 (54.76%)	0.0004
Presence of pericardial effusion, n (%)	290 (18.07%)	39 (33.33%)	0.0001
TAPSE/sPAP ratio [mm/mmHg], median (Q1,Q3)	0.25 (0.18, 0.38)	0.21 (0.15, 0.28)	0.0006
Distance in 6-min walk test [m], median (Q1,Q3)	360 (240, 460)	231 (120, 322.5)	0.0000
Plasma NT-proBNP concentration [pg/mL], median (Q1,Q3)	813.9 (250.55, 2482.5)	2983.5 (1445.75, 5529.75)	0.0000
Use of diuretics, [0/1/2/3/4] n (%)	487 (29.9%) 563 (34.56%) 461 (28.3%) 109 (6.69%) 9 (0.55%)	11 (8.73%) 46 (36.51%) 53 (42.06%) 12 (9.52%) 4 (3.17%)	0.0000
Parenteral use of Treprostinil or Epoprostenol, n (%)	281 (17.25%)	49 (38.89%)	0.0000

**Table 2:** Mean performance metrics for the test set.

Metric	Result	95% Confidence Interval
Accuracy	0.738	0.695–0.783
Sensitivity	0.800	0.636–0.947
Specificity	0.733	0.688–0.779
MCC	0.298	0.195–0.399
ROC-AUC	0.767	0.682–0.843

**Table 3:** Confusion matrix for the test set.

	Predicted		
True		Negative	Positive
	Negative	TN = 239	FP = 87
	Positive	FN = 5	TP = 20

## Data Availability

The data underlying this article will be shared on reasonable request to the BNP-PL steering committee.

## Abbreviations

The following abbreviations are used in this manuscript:

AI	Artificial Intelligence
BNP-PL	Database of Pulmonary Hypertension in the Polish population
CTEPH	Chronic Thromboembolic Pulmonary Hypertension
LASSO	Least Absolute Shrinkage and Selection Operator algorithm
MCC	Matthews Correlation Coefficient
ML	Machine learning
PAH	Pulmonary Arterial Hypertension
ROC-AUC	The Receiver Operating Characteristic—Area Under the Curve
SFE	Sequential Feature Elimination
SHAP	Shapley Additive Explanations
sPAP	Systolic Pulmonary Arterial Pressure
TAPSE	Tricuspid Annular Plane Systolic Excursion
XAI	eXplainable Artificial Intelligence
XGBoost	eXtreme Gradient Boosting
